# Insomnia and caregiver burden in chronic pain patients: A cross-sectional clinical study

**DOI:** 10.1371/journal.pone.0230933

**Published:** 2020-04-02

**Authors:** Masahiro Yamaguchi, Keiko Yamada, Masako Iseki, Yusuke Karasawa, Yasuko Murakami, Tatsuya Enomoto, Nobuko Kikuchi, Satoko Chiba, Atsuko Hara, Keisuke Yamaguchi, Eiichi Inada

**Affiliations:** 1 Department of Pain Medicine, Juntendo University Graduate School of Medicine, Bunkyo-ku, Tokyo, Japan; 2 Medical Affairs, Pfizer Japan, Shibuya-ku, Tokyo, Japan; 3 Department of Anesthesiology and Pain Medicine, Juntendo University Faculty of Medicine, Bunkyo-ku, Tokyo, Japan; 4 Department of Psychology, McGill University, Montreal, Quebec, Canada; Tokyo Metropolitan Institute of Medical Science, JAPAN

## Abstract

Insomnia is a major comorbid symptom of chronic pain and is likely to affect caregiver burden. This cross-sectional study investigated the association between insomnia in chronic pain patients and family caregiver burden. Participants were 60 patients with chronic pain of ≥3 months duration. Demographic and clinical information were collected using the Athens Insomnia Scale (AIS), the Pain Disability Assessment Scale (PDAS), the Hospital Anxiety and Depression Scale (HADS), and a pain intensity numerical rating scale (NRS). Family members who accompanied chronic pain patients to hospital completed the Zarit Burden Interview (ZBI). Univariate regression analysis and multiple regression analysis were conducted to clarify the associations between ZBI scores and total/subscale AIS scores. Covariates were age; sex; pain duration; and scores on the PDAS, HADS anxiety subscale, HADS depression subscale, and NRS. Insomnia was independently associated with ZBI scores [β: 0.27, 95% confidence interval (CI): 0.07–0.52, *p* = 0.001]. Scores on the AIS subscale of physical and mental functioning during the day were significantly associated with ZBI scores (β: 0.32, 95% CI: 0.05–0.59, *p* = 0.007). In conclusion, the findings suggest that in chronic pain patients, comorbid insomnia and physical and mental daytime functioning is associated with family caregiver burden independently of pain duration, pain-related disability, and pain intensity.

## Introduction

Rapid global population aging has led to an increase in the number of people requiring care. In Japan, a public survey of caregiving conducted by the Ministry of Health, Labour and Welfare identified millions of people who receive home-based care and this figure has been increasing annually [1]. In 2001, the main home-based caregivers were partners (25.9%), children (19.9%), and partners of children (22.5%) living with care recipients [1]. As the number of older people who need home-based care increases, family caregiver burden also increases. Physical and mental problems in caregivers (e.g., sleep disturbances) are significantly associated with increasing caregiver burden [2–5].

Chronic pain affects general health [6–9] and hinders basic activities, which negatively affects the quality of life of chronic pain patients [10]. Musculoskeletal disorders such as arthritis, bone fractures, and falls are the main causes (21.1%) of long-term care requirements among older Japanese people [11]. Musculoskeletal disorders are also related to chronic pain in older people, and patients with chronic pain experience various comorbidities (e.g., sleep disturbance) [12]. Sleep disturbance (including insomnia) is associated with pain intensity [13–16], disability [13], depression [13,15,16], and anxiety [16]. Although insomnia as a distinct clinical disorder is experienced by 6% to 14% of community residents [17–19], the risk of sleep disturbance (including insomnia) in people with chronic pain is two to five times greater than in people without chronic pain [20–23].

We hypothesized that in chronic pain patients, insomnia (a major comorbidity of chronic pain) would be associated with increased family caregiver burden through an increased need for mental and physical support by family members. To our knowledge, no previous studies have examined the association between insomnia and family caregiver burden in chronic pain patients. We also hypothesized that the insomnia subtype of nighttime awakening (and concomitant pain complaints) in chronic pain patients would increase family caregiver burden by interrupting the sleep of family members. Therefore, we examined the effect of eight insomnia subtypes: sleep induction, nighttime awakening, final awakening earlier than desired, sleep duration, sleep quality, daytime sense of well-being, daytime functioning, and daytime sleepiness [24].

We investigated 1) whether insomnia in chronic pain patients is associated with family caregiver burden and 2) which insomnia subtypes are associated with family caregiver burden.

## Methods

### Study population

**Fig 1:**
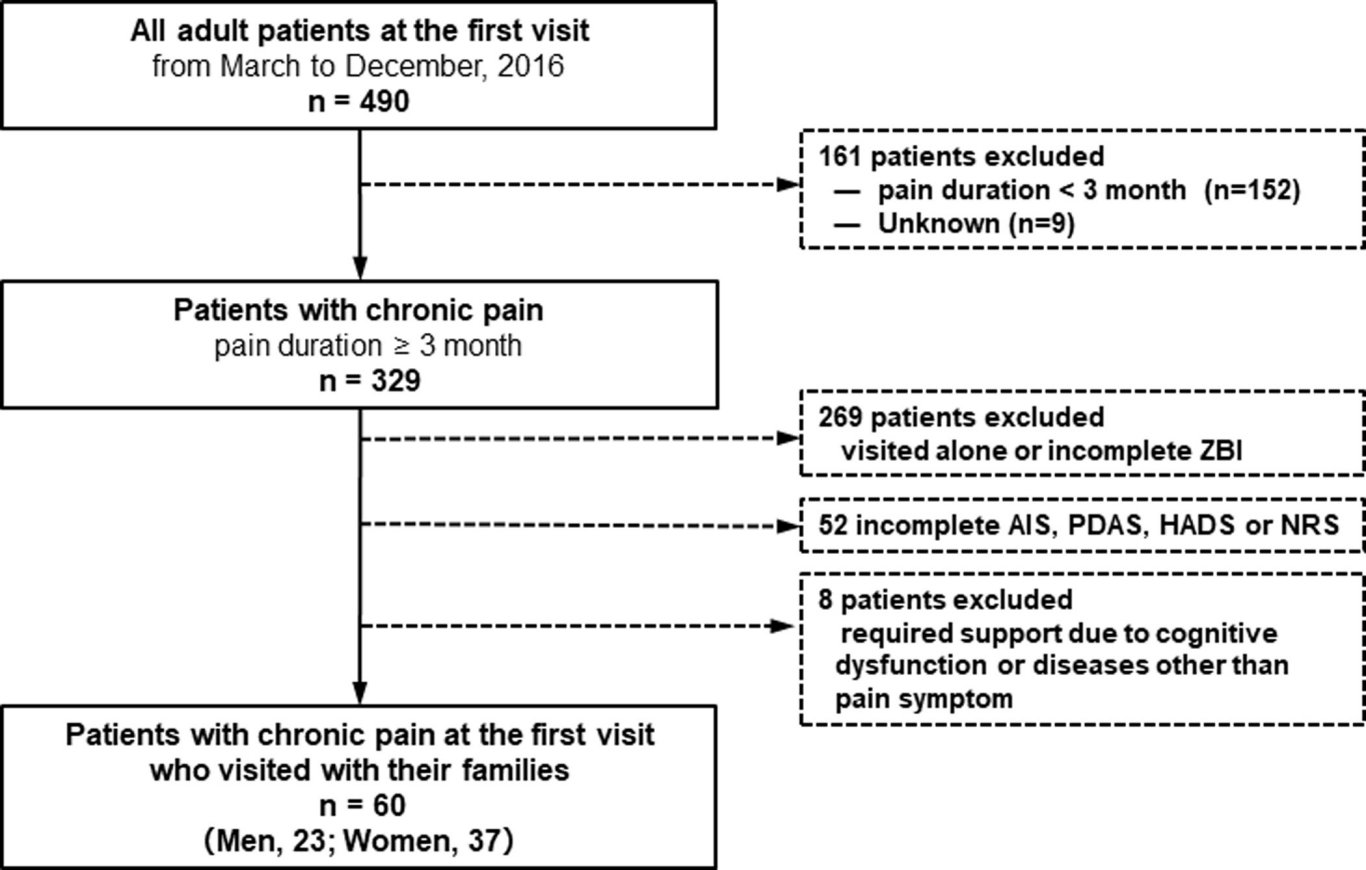
Diagram of the participant selection process.

The participant selection process is shown in Fig 1. From March 2016 to December 2016, 490 outpatients aged 20 years or more visited the Juntendo University Hospital pain clinic for a first visit. Of 490 adult patients, 152 patients who had experienced pain for 3 months and 9 patients with no pain were excluded to focus on chronic pain (pain duration ≥3 months).

Of 329 patients with chronic pain, 269 visited alone or did not complete the Zarit Caregiver Burden Interview (ZBI); 52 did not complete the Athens Insomnia Scale (AIS), the Pain Disability Assessment Scale (PDAS), the Hospital Anxiety and Depression Scale (HADS), or a numerical rating scale (NRS); and 8 required support owing to cognitive dysfunction or diseases other than pain. These patients were excluded.

Data from 60 adult outpatients (23 men and 37 women) with chronic pain who visited with their family and completed the ZBI, AIS, PDAS, HADS, and NRS at the first visit were analyzed in this study.

### Procedure

Patients and their families completed the self-report questionnaires in the waiting room of the at their first visit. A physician who was a specialist of pain medicine classified all participants according to chronic pain diagnosed criteria of the International Statistical Classification of Disease and Related Health Problems 11th Revision (ICD-11) [25]. This study was a cross-sectional retrospective chart review. Demographic data, medical history, duration of pain, and diagnosis were retrospectively collected from participants’ medical records.

### Main measures

#### Caregiving burden

The Zarit Burden Interview (ZBI) [26–28] was originally developed to assess level of subjective burden in caregivers of people with dementia. The ZBI contains 22 items that measure the impact of disease on the physical health, emotional health, social activities, and financial status of caregivers. Each item has five response options ranging from 0 (never) to 4 (nearly always); the maximum ZBI score is 88, and higher scores indicate greater caregiver burden. The ZBI is considered a generic measure of caregiving burden [29,30].

#### Insomnia

The Athens Insomnia Scale (AIS) full version is an eight-item self-report questionnaire that measures the intensity of sleep difficulty [24,31] in accordance with the International Statistical Classification of Disease and Related Health Problems 10th Revision (ICD-10) diagnostic criteria. Each item has four response options ranging from 0 to 3; the maximum score is 24 [32]. Patients rate those items of sleep difficulty that they have experienced at least three times a week or more in the previous month. Five AIS items assess sleep induction difficulty, awakening during the night, early morning awakening, total sleep duration, and overall sleep quality. Three items assess consequences of insomnia for the next day: sense of well-being during the day, physical and mental functioning during the day, and sleepiness during the day. The AIS ≥ 8 was defined as insomnia among individuals with chronic pain [33].

#### Disability due to pain

The Pain Disability Assessment Scale (PDAS) [34] assesses pain-related disability and its impact on daily functioning during the previous 7 days. The PDAS comprises 20 items, each of which is rated on a 4-point scale from 0 (“Pain did not interfere with this activity”) to 3 (“Pain completely interfered with this activity”). Total PDAS scores can range from 0 to 60; higher scores indicate a greater degree of disability.

#### Anxiety and depression symptoms

The Hospital Anxiety and Depression Scale (HADS) [35] measures anxiety and depressive symptoms. The HADS comprises 14 items; 7-item subscales in anxiety and 7-item subscales in depression. Each item is rated on a scale from 0 (not at all) to 3 (most of time) according to how the respondent has felt during the previous 7 days. The total possible score range for each subscale is 0 to 21.

#### Pain intensity

A numerical rating scale (NRS) assesses pain intensity [36]. NRS is rated on a score form 0 (“no pain”) to10 (“worst pain”). In this study, participants rated the average intensity of pain they had experienced in the past 24 hours.

### Statistical analysis

To test differences in mean values according to classification of chronic pain, we used analysis of covariance with the Tukey–Kramer method.

Univariate regression analysis was conducted to investigate which variables were association with family caregiver burden. Subsequently, multiple regression analysis was conducted to investigate which of the variables that were significant in the univariate regression predicted family caregiver burden. The dependent variable was ZBI, the explanatory variable was insomnia, and covariates were age (every 1 year), sex, pain duration (months), PDAS score, HADS anxiety score, HADS depression score, and NRS score.

Next, univariate regression analysis was conducted to investigate which subtypes of insomnia were associated with family caregiver burden. Multiple regression analysis was then conducted to investigate which insomnia subtypes that were significant in the univariate regression analysis predicted family caregiver burden. The dependent variable was ZBI and the explanatory variable was score on the eight AIS subscales.

For subgroup analysis, univariate regression analysis was conducted to investigate which variables were associated with family caregiver burden in the chronic neuropathic pain (n = 31) and chronic musculoskeletal pain (n = 11) categories, which were the two largest chronic pain categories in the current study. The aim was to investigate whether the impact of insomnia on family caregiver burden differed according to chronic pain classification.

Statistical analyses were performed using EZR version 1.27 and SAS version 9.4 (SAS Institute Inc., Cary, NC, USA). All statistical inferences were based on a significance level of *p* < 0.05 (two-tailed tests).

### Ethical considerations

This study was approved by the Institutional Review Board for Clinical Research of Juntendo University Hospital (IRB No.17-234). Informed consent for future use of their data for academic purposes was obtained from all participants before they completed the measures. Furthermore, the current retrospective chart review was placed on the Juntendo University Hospital website, and patients had the chance to opt-out and refuse to participate in this research.

## Results

Participant characteristics in the current retrospective chart review are shown in Table 1. There were no significant differences in the mean scale scores according to classification of chronic pain.

**Table 1:** Participant characteristics.

	mean	(SD)												
Age, years	66.9	(15.1)												
	n	(%)												
Men	37	(61.7)												
Women	23	(38.3)												
Pain Duration														
3 months to 5 months	12	(20.0)												
6 months to 11 months	11	(18.3)												
12 months or more	37	(61.7)												
	mean	(SD)												
ZBI of caregivers (0–88)	17.9	(16.8)												
AIS of patients (0–24)	9.4	(5.1)												
PDAS of patients (0–60)	36.8	(15.3)												
HADS anxiety of patients (0–21)	7.8	(3.8)												
HADS depression of patients (0–21)	10.1	(5.1)												
NRS of patients (0–10)	7.0	(1.8)												
			ZBI of caregivers		AIS		PDAS		HADS anxiety		HADS depression		NRS	
Classification of chronic pain according to ICD-11	n	(%)	mean	(SD)	mean	(SD)	mean	(SD)	mean	(SD)	mean	(SD)	mean	(SD)
1. Chronic Primary Pain	7	(11.7)	14.4	(13.8)	10.1	(7.3)	23.0	(17.7)	8.4	(4.0)	10.6	(5.8)	7.9	(1.9)
2. Chronic Cancer Pain	3	(5.0)	8.0	(6.0)	7.3	(10.2)	45.3	(9.3)	11.0	(7.5)	13.0	(6.6)	8.3	(1.5)
3. Chronic Postsurgical and Posttraumatic Pain	6	(10.0)	17.8	(20.2)	10.5	(4.1)	38.7	(14.3)	8.3	(3.6)	12.0	(5.1)	7.3	(0.8)
4. Chronic Neuropathic Pain	31	(51.7)	23.4	(18.4)	9.5	(4.1)	39.1	(13.6)	7.4	(3.6)	10.2	(4.7)	7.0	(1.9)
5. Chronic Headache and Orofacial Pain	1	(1.7)	0.0	-	12.0	-	60.0	-	3.0	-	3.0	-	8.0	-
6. Chronic Visceral Pain	1	(1.7)	22.0	-	18.0	-	53.0	-	6.0	-	7.0	-	8.0	-
7. Chronic Musculoskeletal Pain	11	(18.3)	8.7	(7.9)	7.5	(5.1)	32.0	(15.5)	8.0	(3.2)	8.8	(5.8)	5.9	(1.8)
p for difference			0.16		0.41		0.05		0.58		0.59		0.23	

*Abbreviation*: ZBI; Zarit Burden Interview; AIS; Athens Insomnia Scale, PDAS; Pain Disability Assessment Scale, HADS; Hospital Anxiety and Depression Scale, NRS; Numerical Rating Scale, SD; standard deviation, ICD; International Statistical Classification of Diseases and Related Health Problems.

The P for difference was calculated by the analysis of covariance using the Turkey–Kramer method.

The associations between family caregiver burden and each variable in the univariate and multiple regression analyses are shown in Tables 2 and 3, respectively. The univariate regression showed that age, PDAS scores, insomnia, and HADS depression scores were significant predictors of ZBI scores. Of these significant predictors, the multiple regression showed that insomnia in chronic pain patients significantly predicted family caregiver burden (standardized regression coefficient [β] = 0.27, *p* = 0.001). Pain duration, pain-related disability, and pain intensity were not associated with family caregiver burden.

**Table 2:** Univariate regression analysis examining predictors of Zarit Burden Interview score. (n = 60).

	β (95% CI)	R^2^	F_change_ (df)	p value
**Dependent = ZBI**				
**Age, years**	0.33** (0.09–0.58)	0.11	7.3 (1, 58)	0.009
**Women**	-0.01 (-0.28–0.21)	<0.001	0.01 (1, 58)	0.91
**Duration of pain, months**	-0.06 (-0.32–0.20)	0.004	0.2 (1, 58)	0.65
**Insomnia**^a^	0.41**(0.17–0.65)	0.17	11.8 (1, 58)	0.001
**PDAS**	0.37** (0.12–0.61)	0.13	9.0 (1, 58)	0.004
**HADS Anxiety**	0.07 (-0.19–0.33)	0.01	0.3 (1, 58)	0.59
**HADS Depression**	0.35**(0.10–0.59)	0.12	8.0 (1, 58)	0.006
**NRS**	-0.04 (-0.30–0.22)	0.002	0.1 (1, 58)	0.76

*Abbreviation*: ZBI; Zarit Burden Interview, AIS; Athens Insomnia Scale, PDAS; Pain Disability Assessment Scale, HADS; Hospital Anxiety and Depression Scale, NRS; Numerical Rating Scale, β; standardized regression coefficient, CI; confidence interval.

^a^Insomnia was defined by Athens Insomnia Scale ≥ 8.

**p < 0.01.

**Table 3:** Multiple regression analysis examining predictors of Zarit Burden Interview score. (n = 60).

	β (95% CI)	R^2^_change_	F_change_ (df)	p value
**Dependent = ZBI**		0.28	5.4 (4, 5)	0.001
**Age, year**	0.19 (-0.06–0.44)			
**Insomnia**^a^	0.26* (0.01–0.52)			
**PDAS**	0.13 (-0.14–0.40)			
**HADS depression**	0.18 (-0.07–0.43)			

*Abbreviation*: ZBI: Zarit Burden Interview; AIS: Athens Insomnia Scale; PDAS: Pain Disability Assessment Scale; HADS: Hospital Anxiety and Depression Scale; β: standardized regression coefficient; CI: confidence interval.

^a^Insomnia was defined by Athens Insomnia Scale ≥ 8.

*p <0.05

The insomnia subtypes that predicted family caregiver burden in the univariate and multiple regressions are shown in Tables 4 and 5, respectively. Of the AIS subscales, the univariate regression identified the following significant predictors of caregiver burden (ZBI score): awakening during the night (β = 0.30, *p* = 0.02), final awakening earlier than desired (β = 0.26, *p* = 0.04), and functioning during the day (β = 0.40, *p* = 0.002). Of these predictors, the multiple regression showed that functioning during the day significantly predicted family caregiver burden (β = 0.32, *p* = 0.007).

**Table 4:** Univariate regression analysis examining Athens Insomnia Scale predictors of Zarit Burden Interview score. (n = 60).

AIS subscales	β (95% CI)	R^2^	F_change_ (df)	p value
**Dependent = ZBI**				
**Sleep induction**^b^	0.11 (-0.15–0.37)	0.01	0.7 (1, 58)	0.41
**Awakening during the night**	0.30* (0.05–0.55)	0.09	5.9 (1, 58)	0.02
**Final awakening earlier than desired**	0.26* (0.01–0.51)	0.07	4.4 (1, 58)	0.04
**Total sleep duration**	0.03 (-0.23–0.29)	<0.001	0.1 (1, 58)	0.82
**Overall quality of sleep**^c^	0.25 (-0.01–0.50)	0.06	3.8 (1, 58)	0.06
**Sense of well-being during the day**	0.09 (-0.17–0.36)	0.01	0.5 (1, 58)	0.48
**Functioning during the day**^d^	0.40** (0.16–0.64)	0.16	11.0 (1,58)	0.002
**Sleep during the day**	0.005 (-0.26–0.27)	0.00	0.0 (1, 58)	0.97

*Abbreviation*: AIS; Athens Insomnia Scale, β; standardized regression coefficient, CI; confidence interval.

^b^Sleep induction was defined as time it takes to fall asleep after turning off the lights,

^c^overall quality of sleep was rated regardless of time slept, and.

^d^functioning during the day was defined as physical and mental functioning.

*p < 0.05,

**p < 0.01.

**Table 5:** Multiple regression analyses examining Athens Insomnia Scale predictors of Zarit Burden Interview score. (n = 60).

AIS subscales	β (95% CI)	R^2^_change_	F_change_ (df)	p value
Dependent = ZBI		0.19	4.5 (3, 56)	0.007
Awakening during the night	0.10 (-0.21–0.40)			
Final awakening earlier than desired	0.13 (-0.15–0.41)			
Functioning during the day^d^	0.32* (0.05–0.59)			

*Abbreviation*: β: standardized regression coefficient; CI: confidence interval.

^d^Functioning during the day was defined as physical and mental functioning.

*p < 0.05.

The subgroup analysis showed that for chronic neuropathic pain (n = 31), only insomnia predicted ZBI score (β = 0.43, p < 0.05) (S1 Table). For chronic musculoskeletal pain (n = 11), insomnia did not significantly contribute to ZBI score (S2 Table).

## Discussion

The study results indicated that insomnia in chronic pain patients was associated with family caregiver burden independently of pain duration, pain-related disability, and pain intensity. The insomnia subtype of physical and mental functioning during the day was associated with family caregiver burden. The subgroup analysis of caregiver burden for chronic neuropathic pain and chronic musculoskeletal pain showed that insomnia predicted family caregiver burden only for chronic neuropathic pain.

Generally, support from both healthcare providers and family are important for chronic pain patients to maintain their daily quality of life. However, family members who provide substantial daily support for chronic pain patients experience great physical and mental burden. For example, a previous research study on cancer patient family burden found a mean ZBI score of 23.2 [37]. In the present study, the mean ZBI score for family members of chronic pain patients with insomnia was very similar (22.9). We believe it is an important finding that family members of chronic pain patients with insomnia experience a substantial caregiving burden, as most chronic pain patients require long-term pain treatment, resulting in a concomitant long-term heavy caregiving burden for family caregivers. A heavy caregiving burden is not only a risk for caregiver depressive symptoms, but also a risk for physical abuse against care recipients [38]. In addition, caregiver stress owing to perceived burden may affect mood disorders such as depression and anxiety in patients with chronic pain, subsequently affecting patient insomnia [13–16, 39, 40]. To avoid these risks, family caregiver burden should be reduced. Therefore, we believe it is important to improve insomnia and daily functioning in chronic pain patients as well as to reduce pain.

Contrary to our hypothesis, nighttime awakening was not associated with increased family caregiver burden. It is possible that chronic pain patients with insomnia tend to tolerate pain-related symptoms at night alone, but continuous mental and physical stress from insomnia owing to chronic pain may affect the daytime activities and mood of chronic pain patients. Research is needed on the lifestyle and living environment of chronic pain patients and family members; however, the effect of insomnia on chronic pain patients’ daytime activities and mood may be related to an increased family caregiver burden.

We performed subgroup analyses for two chronic pain categories (chronic neuropathic pain and musculoskeletal pain), as the sample size was too small to compare all pain categories. Only insomnia in patients with chronic neuropathic pain predicted caregiving burden. This suggests that the impact of insomnia on caregiving burden may differ according to chronic pain classification. However, we can draw only limited conclusions from these two subgroup analyses. The results may have been different if there had been more participants with musculoskeletal pain.

There were several limitations in the present study. We cannot assume that improving sleep disturbances in chronic pain patients would reduce caregiving burden. However, any comprehensive chronic pain treatment strategy should take into account the association between insomnia in chronic pain patients and family caregiver burden. Second, this study was conducted in a single hospital and patients were not stratified by different chronic pain diagnoses, as the sample size was small. Third, we did not examine socioeconomic status (e.g., marital status, number of family members, income, education, occupation) of patients and their families, or the medical history of family caregivers who accompanied patients to the pain clinic. Such variables may interact with caregiving burden and insomnia of patients with chronic pain. Further research is needed to clarify these findings by examining caregiving burden in different populations, such as chronic pain patients with neuropathic pain.

## Conclusion

Chronic pain comorbid with insomnia was associated with high family caregiver burden independent of pain severity, pain duration, and pain-related disabilities. Analysis of insomnia subtypes showed that physical/mental functioning during the day was associated with family caregiver burden, suggesting that treatment of insomnia in chronic pain patients indirectly affects family caregivers.

## Supporting information

S1 Table Univariate regression analysis examining predictors of Zarit Burden Interview score among chronic neuropathic pain (n = 31). (PDF)

S2 Table Univariate regression analysis examining predictors of Zarit Burden Interview score among chronic musculoskeletal pain (n = 11). (PDF)
