# Supplementary material for: Insomnia and caregiver burden in chronic pain patients: A cross-sectional clinical study
Source: PLoS One. 2020 Apr 2;15(4):e0230933. doi: 10.1371/journal.pone.0230933 (PMC7117677; doi:10.1371/journal.pone.0230933)
Supplement: S1 Table — (PDF) [file pone.0230933.s001.pdf]

S1 Table. Univariate regression analysis examining predictors of Zarit Burden Interview score among chronic neuropathic pain (n=31)

	β (95% CI)	R^2	$F_{change}(df)$	*p value*
Dependent=ZBI				
Age, years	0.23 (-0.14 – 0.60)	0.05	1.6 (1, 29)	0.21
Women	0.06 (-0.32 – 0.44)	0.004	0.1 (1, 29)	0.75
Duration of pain, months	0.34 (-0.17 – 0.65)	0.120	3.9 (1, 29)	0.06
Insomnia	0.43* (0.09 – 0.77)	0.18	6.5 (1, 29)	0.02
PDAS	0.32 (-0.04 – 0.68)	0.10	3.3 (1, 29)	0.08
HADS Anxiety	0.05 (-0.33 – 0.43)	0.00	0.1 (1, 29)	0.78
HADS Depression	0.32 (-0.04 – 0.68)	0.10	3.3 (1, 29)	0.08
NRS	-0.14 (-0.51 – 0.24)	0.020	0.5 (1, 29)	0.47

Abbreviation: ZBI; Zarit Burden Interview, PDAS; Pain Disability Assessment Scale, HADS; Hospital Anxiety and Depression Scale, NRS; Numerical Rating Scale, β; standardized regression coefficient, CI; confidence interval.

Insomnia was defined by Athens Insomnia Scale ≥ 8. *$p < 0.05$.
