# Supplementary material for: Insomnia and caregiver burden in chronic pain patients: A cross-sectional clinical study
Source: PLoS One. 2020 Apr 2;15(4):e0230933. doi: 10.1371/journal.pone.0230933 (PMC7117677; doi:10.1371/journal.pone.0230933)
Supplement: S2 Table — (PDF) [file pone.0230933.s002.pdf]

S2 Table. Univariate regression analysis examining predictors of Zarit Burden Interview score among chronic musculoskeletal pain (n=11)

	β (95% CI)	R^2	$F_{change}(df)$	*p value*
Dependent=ZBI				
Age, years	0.04 (-0.71 – 0.79)	0.002	0.01 (1, 29)	0.91
Women	0.28 (-0.45 – 1.00)	0.080	0.8 (1, 29)	0.41
Duration of pain, months	0.13 (-0.62 – 0.88)	0.020	0.2 (1, 29)	0.70
Insomnia	0.45 (-0.23 – 1.12)	0.20	2.2 (1, 29)	0.17
PDAS	0.28 (-0.44 – 1.01)	0.08	0.8 (1, 29)	0.40
HADS Anxiety	0.54 (-0.09 – 1.17)	0.29	3.7 (1, 29)	0.09
HADS Depression	0.53 (-0.11 – 1.17)	0.28	3.5 (1, 29)	0.09
NRS	0.26 (-0.47 – 0.99)	0.070	0.6 (1, 29)	0.44

Abbreviation: ZBI; Zarit Burden Interview, PDAS; Pain Disability Assessment Scale, HADS; Hospital Anxiety and Depression Scale, NRS; Numerical Rating Scale, β; standardized regression coefficient, CI; confidence interval.

Insomnia was defined by Athens Insomnia Scale ≥ 8.
